# Population-based prevalence and incidence estimates of primary discoid lupus erythematosus from the Manhattan Lupus Surveillance Program

**DOI:** 10.1136/lupus-2019-000344

**Published:** 2019-10-30

**Authors:** Peter Izmirly, Jill Buyon, H Michael Belmont, Sara Sahl, Isabella Wan, Jane Salmon, Anca Askanase, Joan M Bathon, Laura Geraldino-Pardilla, Yousaf Ali, Ellen Ginzler, Chaim Putterman, Caroline Gordon, Charles Helmick, Hilary Parton

**Affiliations:** 1 Division of Rheumatology, Department of Medicine, New York University School of Medicine, New York City, New York, USA; 2 Department of Pediatrics, Harbor-University of California Medical Center, Los Angeles, California, USA; 3 Division of Rheumatology, Department of Medicine, Hospital for Special Surgery, Weill Cornell Medical College, New York City, New York, USA; 4 Division of Rheumatology, Department of Medicine, Columbia University College of Physicians & Surgeons, New York City, New York, USA; 5 Division of Rheumatology, Department of Medicine, Icahn School of Medicine at Mount Sinai, New York City, New York, USA; 6 Division of Rheumatology, Department of Medicine, State University of New York Downstate College of Medicine, Brooklyn, New York, USA; 7 Division of Rheumatology, Department of Medicine, Albert Einstein College of Medicine, Bronx, New York, USA; 8 Rheumatology Research Group, Institute of Inflammation and Ageing, College of Medical and Dental Sciences, University of Birmingham, Birmingham, UK; 9 Arthritis Program, Centers for Disease Control and Prevention, Atlanta, Georgia, USA; 10 New York City Department of Health and Mental Hygiene, Long Island City, New York, USA

**Keywords:** epidemiology, Discoid Lupus Erythematosus, Registry

## Abstract

**Objective:**

Epidemiological data for primary discoid lupus erythematosus (pDLE) remain limited, particularly for racial/ethnic populations in the USA. The Manhattan Lupus Surveillance Program (MLSP) is a population-based retrospective registry of cases with SLE and related diseases including pDLE in Manhattan and was used to provide estimates of the prevalence and incidence of pDLE across major racial/ethnic populations.

**Methods:**

MLSP cases were identified from rheumatologists, hospitals and population databases. Two case definitions were used for pDLE: the primary case definition which was any physician diagnosis found in the chart and a secondary case definition which was limited to cases diagnosed by a rheumatologist and/or dermatologist. Rates among Manhattan residents were age-adjusted, and capture–recapture analyses were conducted to assess case under-ascertainment.

**Results:**

Based on the primary definition, age-adjusted overall prevalence and incidence rates of pDLE among Manhattan residents were 6.5 and 0.8 per 100 000 person-years, which increased to 9.0 and 1.3 after capture–recapture adjustment. Prevalence and incidence rates were approximately two and six times higher, respectively, among women compared with men (p<0.0001). Higher prevalence was also found among non-Latino blacks (23.5) and Latinos (8.2) compared with non-Latino whites (1.8) and non-Latino Asians (0.6) (p<0.0001). Incidence was highest among non-Latino blacks (2.4) compared with all other racial/ethnic groups. Similar relationships were observed for the secondary case definition.

**Conclusion:**

Data from the MLSP provide epidemiological estimates for pDLE among the major racial/ethnic populations in the USA and reveal disparities in pDLE prevalence and incidence by sex and race/ethnicity among Manhattan residents.

## Introduction

Discoid lupus erythematosus (DLE) is one of the most common cutaneous manifestations of SLE, with recent population-based studies estimating it occurs in approximately 12.4%–15.0% of incident SLE cases^1–3^ and in approximately 16.6%–24.3% of prevalent cases.^1 3 4^ The highest rates of DLE in patients with SLE are seen among black patients.^1–4^ DLE is associated with considerable morbidity, as it tends to occur on the face, scalp and ears, and is associated with scarring and permanent alopecia.^5 6^ DLE can also occur in the absence of SLE, referred to as primary DLE (pDLE). The extant epidemiological data on pDLE remain limited with few published estimates for the general population and scant data for racial/ethnic populations in the USA.^7^

The Manhattan Lupus Surveillance Program (MLSP) is one of five Centers for Disease Control and Prevention (CDC)–funded population-based registries composed of patients with SLE and related diseases.^1–4 8^ Recently, the Georgia Lupus Registry (GLR) reported an overall age-adjusted incidence of pDLE of 3.7 per 100 000 person-years, with a black:white ratio of 5.3.^7^ We used MLSP data to provide estimates of the prevalence and incidence of pDLE during 2007 and 2007–2009, respectively, in Manhattan which is characterised by a more diverse population (non-Latino black, Latino, non-Latino Asian, non-Latino white) than the GLR.

## Methods

### Manhattan Lupu*s* Surveillance Program

Details on the MLSP have been previously reported.^1 9^ In brief, medical records were reviewed under the health surveillance exemption to HIPAA privacy rules (45 CFR § 164.512(b)) and as authorised by New York City Charter Sections 556(c)(2) and (d)(2) with no potential cases being contacted for this project. The MLSP was deemed surveillance that did not require institutional review board (IRB) review by IRBs at the CDC, the New York City Department of Health and Mental Hygiene (DOHMH) and the New York University School of Medicine. Additional IRB applications were completed and submitted to independent case-finding sources when requested. The DOHMH IRB reviewed and approved secondary analyses on a de-identified dataset including the analyses presented here.

The surveillance period for the MLSP was 1 January 2007 through 31 December 2009 with Manhattan being chosen for reasons previously described.^1^ Based on 2010 US Census data, there were 1 585 873 persons residing in Manhattan (48% non-Latino white, 25% Latino, 13% non-Latino black, 11% non-Latino Asian).^10^

### Case ascertainment, data collection and quality control of data entry

Case-finding sources for the MLSP included rheumatologists’ practices (including paediatric rheumatologists), hospitals (rarely including associated dermatology clinics), and administrative hospitalisation discharge and death registry databases.^1^ Sources were queried retrospectively to identify patients who lived in Manhattan with the following International Classification of Disease Ninth Revision Clinical Modification (ICD-9CM) billing codes: 710.0 (SLE), 695.4 (DLE), 710.8 (other specified connective tissue disease), 710.9 (unspecified connective tissue disease) and 710.2 (Sicca syndrome, which is used for Sjögren’s syndrome). Charts for every patient who had one of the respective ICD-9CM codes and was confirmed to live in Manhattan were fully abstracted and final diagnosis and date of diagnosis were coded. In addition, the type of physician (rheumatologist, dermatologist) making the diagnosis was also coded. Abstraction was performed by trained abstractors with medical degrees who underwent extensive training and routine quality assurance; abstraction was completed in 90.5% of hospitals and 75.8% of rheumatologists’ practices.^1^

### Case definitions

Our primary case definition was any statement by a physician that the patient carries a diagnosis of pDLE found in the chart. The diagnosis could be stated by a rheumatologist (both as inpatient or outpatient) or dermatology note (if seen as an inpatient, or if a consult note was found in a rheumatologist’s chart, or if found in a hospital-associated dermatology clinic). In addition, pDLE would be included if listed under medical history if a patient was admitted for a completely different reason. Our primary case definition was purposefully broad in an effort to capture as many cases as possible to get an estimate of the burden of the disease.

Our more restrictive secondary definition required evidence that the diagnosis was stated by a rheumatologist or dermatologist. Given the MLSP did not approach dermatology practices and only rarely had access to hospital-associated dermatology clinics as a case-finding source, we did not want to further underestimate the burden of DLE by also requiring evidence of a biopsy, which is not always performed.^7^ Thus, we did not require evidence of a compatible diagnostic biopsy for either definition. Cases that met American College of Rheumatology (ACR) Classification criteria for SLE^11 12^ were not considered to be pDLE cases. In addition, we excluded any case that carried a final diagnosis of SLE based on a rheumatologist’s note only and also had evidence of DLE but did not fulfil ACR criteria.

### Statistical analysis

Prevalent cases were new or existing pDLE cases residing in Manhattan from 1 January to 31 December 2007. Incident cases were those residing in Manhattan and first diagnosed with pDLE from 1 January 2007 through 31 December 2009. Denominators were calculated from DOHMH intercensal population estimates for Manhattan.^10^

Rates overall, by sex and by race/ethnicity were calculated per 100 000 person-years and age-adjusted to the USA 2000 standard population.^13^ Due to small counts, we calculated CIs using the gamma method.^14^ Data on race and Latino ethnicity were collected separately, but this information was used to assign cases into five mutually exclusive race/ethnicity categories: Latino, non-Latino white, non-Latino black, non-Latino Asian and non-Latino other (including more than one race). Differences by sex and race/ethnicity were assessed using χ^2^ tests or Fisher’s exact tests. If a significant difference was found by race/ethnicity, pairwise differences were then evaluated using z-tests assuming the Poisson distribution and statistical significance at 0.05, with Bonferroni correction to 0.008. Capture–recapture analyses were performed^15 16^ to estimate case under-ascertainment,^1^ with log-linear models fit separately for incident and prevalent cases by race/ethnicity. We fit various models that addressed potential violation of the homogeneity assumption of capture probability and identified the best-fitting model using the Akaike information criterion. Then we used these model estimates to calculate revised prevalence and incidence rates.^15 16^

Frequency and location of discoid rash information was described only among cases meeting the secondary definition, as these cases with evidence of diagnosis by a rheumatologist or dermatologist were more likely to have accompanying descriptive evidence in the record.

All analyses were completed using SAS V.9.4 (SAS Institute, Cary, North Carolina, USA) except for capture–recapture analysis that was completed using RStudio Server with R V.3.5.2 (R Foundation for Statistical Computing, Vienna, Austria).

## Results

### Prevalence rates for DLE

Using the primary case definition, a total of 110 cases had a diagnosis of pDLE, with most (71) having evidence of a diagnosis by a rheumatologist or dermatologist (table 1). The crude and age-adjusted prevalence of pDLE by this definition were 7.0 (95% CI 5.7 to 8.3) and 6.5 (95% CI 5.2 to 7.7) per 100 000 person-years (table 2). Age-adjusted rates were approximately two times higher among women compared with men (p<0.0001). The age-adjusted prevalence of pDLE also differed by race/ethnicity (p<0.0001) and was significantly higher among non-Latino blacks (23.5) and Latinos (8.2) compared with non-Latino whites (1.8) and non-Latino Asians (0.6). Capture–recapture estimated an additional 9.8 cases of pDLE, indicating that 8.2% of cases may have been missed. With this adjustment, the overall prevalence of pDLE increased to 9.0 (95% CI 7.0 to 11.0) and among non-Latino blacks increased to 34.2. (95% CI 30.3 to 38.1). Using the secondary (more restrictive) case definition for pDLE lowered the crude and age-adjusted prevalence to 4.5 (95% CI 3.5 to 5.7) and 4.2 (95% CI 3.3 to 5.3) per 100 000 person-years (table 2). Similar disparities were seen, with higher rates among women compared with men (p=0.0006) as well as higher rates among non-Latino blacks and Hispanics (p<0.0001, table 2).

**Table 1:** Evidence of a diagnosis of primary discoid lupus erythematosus among prevalent and incident cases by physician type

Primary case definition	Prevalence n=110	Incidence n=41
No rheumatologist, dermatologist stated the final diagnosis	37	15
One or more rheumatologists stated the final diagnosis	21	10
One or more dermatologists stated the final diagnosis	32	8
A rheumatologist and/or dermatologist stated the final diagnosis	18	8
A pathologist stated the final diagnosis	2	0

**Table 2:** Crude and adjusted prevalence rates of primary discoid lupus erythematosus (pDLE) among Manhattan residents, 2007, overall and by sex and race/ethnicity

Crude rate (95% CI)	Age-adjusted rate (95% CI)	χ^2^ p value	Capture–recapture adjusted rate
Primary definition pDLE—any MD diagnosis				
Total	7.0 (5.7 to 8.3)	6.5 (5.2 to 7.7)		9.0 (7.0 to 11.0)
Male	3.9 (2.6 to 5.6)	3.7 (2.4 to 5.3)	<0.0001	
Female	9.7 (7.7 to 12.0)	8.7 (6.9 to 10.9)		
Race/ethnicity			<0.0001*	
Non-Latino white	2.1 (1.2 to 3.4)	1.8 (1.0 to 2.9)		2.5 (2.0 to 2.9)
Non-Latino black	25.2 (18.9 to 32.8)	23.5 (17.6 to 30.7)		34.2 (30.3 to 38.1)
Latino	7.8 (5.4 to 11.1)	8.2 (5.6 to 11.6)		10.7 (9.3 to 12.0)
Non-Latino Asian	0.6 (0.0 to 3.2)	0.6 (0.0 to 3.5)		0.9 (0.0 to 1.7)
	
Secondary definition pDLE—rheumatologist or dermatologist diagnosis				
Total	4.5 (3.5 to 5.7)	4.2 (3.3 to 5.3)		6.6 (3.2 to 10.1)
Male	2.6 (1.5 to 4.0)	2.4 (1.4 to 3.8)	0.0006	
Female	6.2 (4.6 to 8.1)	5.7 (4.2 to 7.5)		
Race/ethnicity			<0.0001*	
Non-Latino white	0.9 (0.4 to 1.9)	0.8 (0.3 to 1.6)		1.6 (−0.6 to 3.8)
Non-Latino black	18.2 (12.9 to 24.9)	17.2 (12.2 to 23.5)		25.8 (21.2 to 30.4)
Latino	4.7 (2.8 to 7.3)	4.9 (2.9 to 7.6)		7.7 (4.5 to 11.0)
Non-Latino Asian	0.6 (0.0 to 3.2)	0.6 (0.0 to 3.5)		0.9 (0.0 to 1.7)
	

Cases exclude those meeting American College of Rheumatology criteria for SLE.

Rates are per 100 000 Manhattan residents. Denominator data are based on 2007–2009 intercensal population estimates from the New York City Department of Health and Mental Hygiene Bureau of Epi Services (2000–2014 files).

Data are age-adjusted to the US 2000 Standard Population.

Cases were assigned to one of five mutually exclusive race/ethnicity categories: non-Latino white, non-Latino black, non-Latino Asian, Latino and non-Latino other. Non-Latino cases identified with more than one race were categorised as non-Latino other; rates were not calculated for this group.

*Non-Latino blacks differed from non-Latino whites, Latinos and non-Latino Asians. Latinos also differed from non-Latino whites and non-Latino Asians. Non-Latino whites did not significantly differ from non-Latino Asians.

The average ages (±SD) among women and men in 2007 meeting the primary case definition were 53.5 (±17.5) and 40.4 (±13.6) years, respectively. The average age of pDLE cases was highest among non-Latino whites (60.6±19.3) followed by Latinos (53.8±18.0) and non-Latino blacks (45.0±15.1). Age-specific prevalence rates were higher among cases aged 40–59 (7.7, 95% CI 5.3 to 10.9) and 60 and older (6.4, 95% CI 3.8 to 10.1) compared with those aged 20–39 (3.2, 95% CI 1.9 to 4.9).

Among prevalent cases meeting the secondary case definition, discoid rash was localised above the neck for 39.4% and generalised for 28.2%. Almost one-third (32.4%) of cases had no further information available on rash location. Biopsy confirmation of pDLE was rare, with evidence found for only 2.8% of prevalent pDLE cases. ANA results were available in 81.7% of cases, and almost half of these were positive (44.8%).

### Incidence rates for DLE

Based on the primary case definition, we identified 41 incident cases diagnosed with pDLE, with evidence of a diagnosis by a rheumatologist or dermatologist for most (26) (table 3). The overall crude and age-adjusted pDLE incidence rates were 0.9 (95% CI 0.6 to 1.2) and 0.8 (95% CI 0.6 to 1.1) per 100 000 person-years. Age-adjusted rates differed by sex and were approximately four times higher for women compared with men (p≤0.0002). The incidence of pDLE differed by race/ethnicity (p<0.0001) with the highest age-adjusted rates among non-Latino blacks (2.3) followed by Latinos (1.0), non-Latino Asians (0.6) and non-Latino whites (0.3). Based on capture–recapture analysis, we estimated an additional 11.4 cases of pDLE, indicating that 21.8% of cases may have been missed. With capture–recapture adjustment, the overall incidence of DLE increased to 1.3 (95% CI 0.6 to 2.0) and among non-Latino blacks increased to 4.0 (95% CI 2.1 to 5.8). With the secondary case definition, the overall crude and age-adjusted pDLE incidence rates were lower, at 0.5 (95% CI 0.4 to 0.8) and 0.5 (95% CI 0.3 to 0.7) per 100 000 person-years. Disparities were again seen by sex, with rates among women approximately eight times higher than among men (p=0.0002), and race/ethnicity, with higher rates among non-Latino blacks (p<0.0001).

**Table 3:** Crude and adjusted incidence rates of primary discoid lupus erythematosus (pDLE) among Manhattan residents, 2007–2009, overall and by sex and race/ethnicity

	Crude rate (95% CI)	Age-adjusted rate (95% CI)	χ^2^ p value	Capture–recapture adjusted rate
Primary definition pDLE—any MD diagnosis				
Total	0.9 (0.6 to 1.2)	0.8 (0.6 to 1.1)		1.3 (0.6 to 2.0)
Male	0.3 (0.1 to 0.7)	0.3 (0.1 to 0.6)	<0.0001	
Female	1.4 (0.9 to 1.9)	1.3 (0.9 to 1.8)		
Race/ethnicity			<0.0001*	
Non-Latino white	0.3 (0.1 to 0.6)	0.3 (0.1 to 0.6)		0.4 (0.3 to 0.4)
Non-Latino black	2.4 (1.3 to 3.9)	2.3 (1.3 to 3.8)		4.0 (2.1 to 5.8)
Latino	1.0 (0.5 to 1.7)	1.0 (0.5 to 1.7)		1.5 (1.0 to 2.0)
Non-Latino Asian	0.6 (0.1 to 1.7)	0.6 (0.1 to 1.7)		1.1 (-0.5 to 2.7)
	
Secondary definition pDLE—rheumatologist or dermatologist diagnosis				
Total	0.5 (0.4 to 0.8)	0.5 (0.3 to 0.7)		0.9 (0.2 to 1.5)
Male	0.1 (0.0 to 0.4)	0.1 (0.0 to 0.3)	0.0002	
Female	0.9 (0.6 to 1.4)	0.8 (0.5 to 1.3)		
Race/ethnicity			<0.0001†	
Non-Latino white	0.2 (0.1 to 0.5)	0.2 (0.0 to 0.4)		0.2 (0.1 to 0.3)
Non-Latino black	1.9 (1.0 to 3.3)	1.8 (0.9 to 3.2)		3.2 (1.8 to 4.6)
Latino	0.5 (0.2 to 1.1)	0.5 (0.2 to 1.0)		0.9 (-0.1 to 1.8)
Non-Latino Asian	0.6 (0.1 to 1.7)	0.6 (0.1 to 1.7)		0.9 (-0.5 to 2.3)
	

Cases exclude those meeting American College of Rheumatology criteria for SLE.

Rates are per 100 000 Manhattan residents. Denominator data are based on 2007–2009 intercensal population estimates from the New York City Department of Health and Mental Hygiene Bureau of Epi Services (2000–2014 files).

Data are age-adjusted to the US 2000 Standard Population.

Cases were assigned to one of five mutually exclusive race/ethnicity categories: non-Latino white, non-Latino black, non-Latino Asian, Latino and non-Latino other. Non-Latino cases identified with more than one race were categorised as non-Latino other; rates were not calculated for this group.

*Non-Latino blacks differed from non-Latino whites and non-Latino Asians. Latinos also differed from non-Latino whites and non-Latino Asians.

†Non-Latino blacks differed from non-Latino whites and Latinos.

The average age (±SD) at diagnosis among incident pDLE cases meeting the primary case definition was 45.7 (±16.3) years among women and 40.6 (±20.70) years among men. Average age (±SD) at diagnosis was highest among non-Latino whites (63.4±24.1) followed by Latinos (45.8±16.6), non-Latino Asians (45.3±8.5) and non-Latino blacks (36.7±8.6). Age-specific rates suggested a similar pattern, with a higher rate among those aged 40–59, but numbers were too small to provide reliable estimates.

Among incident cases meeting the secondary case definition, 50.0% had discoid rash located above the neck and 11.5% had generalised discoid rash. Over one-third (38.5%) had no further location information available. Among cases with ANA results available (84.6%), 45.5% were positive. As with prevalent cases, biopsy confirmation of pDLE was rare, with evidence found for only 3.8% of incident cases.

## Discussion

Our analysis of the MLSP dataset provides prevalence and incidence estimates of pDLE among Manhattan residents overall and for the major racial/ethnic populations in the USA. Based on our primary case definition, the age-adjusted prevalence and incidence of pDLE in Manhattan were 6.5 (95% CI 5.2 to 7.7) and 0.8 (95% CI 0.6 to 1.1) per 100 000 person-years. Capture–recapture adjustment increased prevalence and incidence rates by 38.5% and 62.5%, respectively. Prevalence of pDLE was significantly higher among non-Latino blacks and Latinos compared with non-Latino Asians and non-Latino whites. The incidence of pDLE was higher among non-Latino blacks compared with the other racial/ethnic groups. These racial disparities mirrored the pattern of DLE seen among SLE cases, with non-Latino black SLE cases having the highest percentage of DLE.^1^

There are limited studies on the epidemiology of pDLE and most focus on various forms cutaneous lupus erythematosus (CLE), including pDLE. A US-based study from Olmsted County, Minnesota, reported a population-based prevalence estimate for CLE of 70.4 per 100 000.^17^ The same study provided an incidence rate of CLE at 4.2 per 100 000. More than half (55%) of the incident cases were reported to have pDLE, suggesting a higher incidence of pDLE than we found in our analysis. Several other studies have reported incidence rates of pDLE, including a study from Sweden and one from French Guiana.^17 18^ The Swedish study provided a pDLE rate of 3.2 per 100 000^18^ while the study from French Guiana, with a predominantly African population, reported an annual chronic CLE incidence of 2.6 per 100 000 population (of which 90% were pDLE).^19^ In line with our findings, both the Minnesota study as well as the Swedish study found that prevalent and incident pDLE were higher among women compared with men and most common in middle-aged women.^7 17 18^ The Minnesota study also reported rates of CLE by race/ethnicity,^17^ but these rates are not comparable given the predominantly white population (90.3%) included in that analysis.

Recently, the Georgia Lupus Registry (GLR), one of the five funded CDC registries, published their incidence estimates of primary chronic CLE and pDLE in a predominantly white and black population from Fulton and DeKalb counties in Georgia.^7^ The GLR age-adjusted and capture–recapture adjusted estimates of pDLE incidence were 3.7 (95% CI 3.2 to 4.3) and 4.0 (95% CI 3.5 to 4.7) per 100 000 person-years. In line with our findings, the GLR also found higher rates among blacks compared with whites and among women compared with men. However, the GLR did not present estimates for other racial/ethnic groups, and to our knowledge, there are no other population-based studies that present pDLE findings among more diverse populations.

Although the GLR and MLSP used similar methods, there were important differences that likely contributed to differences in incidence findings. The GLR approached both dermatologists and dermatopathology laboratories, which were not approached by the MLSP. For the GLR, those case finding sources yielded 70 (36.8%) cases, with 27 cases that were only found in dermatology practices and 43 that were only found in dermatopathology laboratories with chronic CLE that were not captured by other case finding sources.^7^ There were 25 dermatology practices in the GLR catchment area, while there were approximately 350 identified within Manhattan during the MLSP planning. Given our primary focus on SLE and limited resources available to abstract from such a large number of physicians, the MLSP did not approach dermatology practices. This is an obvious limitation to a study focused on capturing a primary cutaneous manifestation of lupus and likely resulted in an underestimate of the MLSP incidence and prevalence estimates of pDLE. In addition, our estimates are biased towards cases requiring hospitalisation or rheumatology evaluation, which may also account for the low percentage of biopsies available from our case finding sources. The low percentage of biopsies found in the charts does not necessarily imply that there was a low rate of biopsies done by physicians treating patients in the MLSP catchment area. Rather this finding reflects the fact that biopsy results were not readily identified in the charts using the MLSP methodology, likely due to the fact that dermatology offices were not recruited to this study. Including cases that were only found through hospitalisation or by a rheumatologist may have biased our case finding towards sicker cases that may have been in the early stages of SLE even if they did not fulfil ACR criteria for SLE. Thus, patients with DLE in evolution towards SLE would be considered in this study as pDLE. There were several limitations regarding the development of the MLSP that have been previously acknowledged and could have accounted for under-ascertainment of prevalent and incident cases.^1 9^ Those included lack of participation from one quarter of rheumatologists and two hospitals in the catchment area, including the Veteran’s Administration Hospital, which may have specifically under-identified men diagnosed with pDLE. In addition, cases would have been missed if they lived in Manhattan but sought care in other boroughs or a neighbouring state. Additional limitations of the MLSP resulted from the tremendous differences across medical systems of case-finding sources and abstracting several years after the surveillance period.^1 9^ Additional limitations pertain to assigning race and ethnicity based on administrative and medical records which have been previously described.^1 9^

Despite these limitations, our analyses benefited from the design and composition of the MLSP, a population-based registry with a diverse catchment population.^1^ This design allowed us to estimate rates of DLE among a larger group of racial/ethnic categories than have been previously reported. The partnership with the DOHMH allowed us to collect information from several case-finding sources and facilitated more complete clinical information on many cases. In addition, capture–recapture analyses were conducted to estimate missed cases. Finally, our abstractors all had medical backgrounds, which helped during training and provided an advantage during extensive review of medical records to identify criteria and manifestations of SLE.^1^

In conclusion, data from our large population-based registry provide epidemiological estimates for pDLE for the major racial/ethnic populations in the USA and reveal substantial disparities by sex and race/ethnicity in pDLE among Manhattan residents. These findings add support to the limited existing epidemiology of this understudied disease, confirming evidence of similar disparities among patients with SLE with DLE and suggesting non-Latino blacks are disproportionately affected whether they have the systemic or primary form.
