# Correction: *Population-based prevalence and incidence estimates of primary discoid lupus erythematosus from the Manhattan Lupus Surveillance Program*

**DOI:** 10.1136/lupus-2019-000344corr1

**Published:** 2019-11-14

Izmirly P, Buyon J, Belmont HM, *et al*. Population-based prevalence and incidence estimates of primary discoid lupus erythematosus from the Manhattan Lupus Surveillance Program. *Lupus Science & Medicine* 2019;6:e000344. doi: 10.1136/lupus-2019-000344

The published version misspelled co-author’s name as Yusaf Ali. The correct name should be Yousaf Ali.

